# Brain Branched-Chain Amino Acids in Maple Syrup Urine Disease: Implications for Neurological Disorders

**DOI:** 10.3390/ijms21207490

**Published:** 2020-10-11

**Authors:** Jing Xu, Youseff Jakher, Rebecca C. Ahrens-Nicklas

**Affiliations:** 1 Division of Human Genetics, Children’s Hospital of Philadelphia, Philadelphia, PA 19104, USA; xuj7@email.chop.edu; 2 Department of Chemistry, University of Pennsylvania, Philadelphia, PA 19104, USA; youseffj@sas.upenn.edu; 3 Department of Pediatrics, Perelman School of Medicine at the University of Pennsylvania, Philadelphia, PA 19104, USA

**Keywords:** maple syrup urine disease, branched-chain amino acid, amino acid metabolism, metabolic disorder, neurological disorder

## Abstract

Maple syrup urine disease (MSUD) is an autosomal recessive disorder caused by decreased activity of the branched-chain α-ketoacid dehydrogenase complex (BCKDC), which catalyzes the irreversible catabolism of branched-chain amino acids (BCAAs). Current management of this BCAA dyshomeostasis consists of dietary restriction of BCAAs and liver transplantation, which aims to partially restore functional BCKDC activity in the periphery. These treatments improve the circulating levels of BCAAs and significantly increase survival rates in MSUD patients. However, significant cognitive and psychiatric morbidities remain. Specifically, patients are at a higher lifetime risk for cognitive impairments, mood and anxiety disorders (depression, anxiety, and panic disorder), and attention deficit disorder. Recent literature suggests that the neurological sequelae may be due to the brain-specific roles of BCAAs. This review will focus on the derangements of BCAAs observed in the brain of MSUD patients and will explore the potential mechanisms driving neurologic dysfunction. Finally, we will discuss recent evidence that implicates the relevance of BCAA metabolism in other neurological disorders. An understanding of the role of BCAAs in the central nervous system may facilitate future identification of novel therapeutic approaches in MSUD and a broad range of neurological disorders.

## 1. Introduction

### 1.1. Branched-Chain Amino Acid Metabolism and Maple Syrup Urine Disease

**Figure 1:**
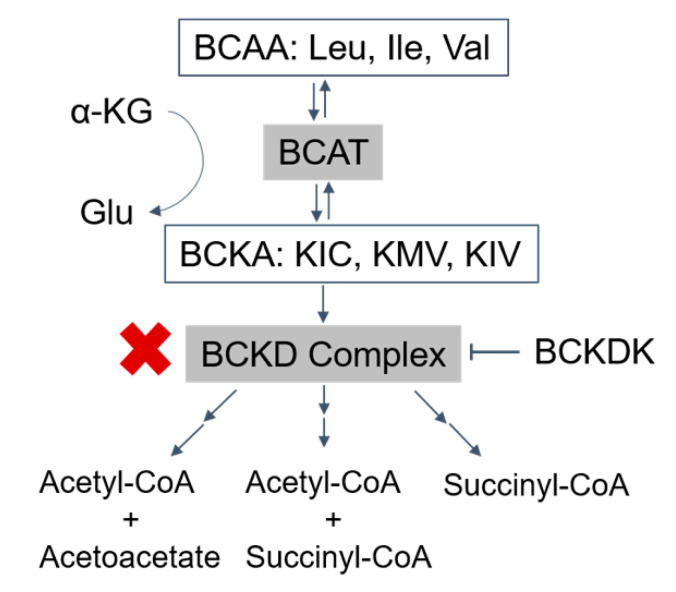
Catabolism pathways of branched-chain amino acids (BCAAs): the BCAAs are transmitted by branched-chain amino acid transaminase (BCAT) to form branched-chain α-ketoacids (BCKAs) (α-ketoisocaproate (KIC), α-keto-β-methylvalerate (KMV), and α-ketoisovalerate (KIV)), which are irreversibly oxidized by the BCKD complex. The final products, acetyl-CoA, acetoacetate, and succinyl-CoA, are produced after a series of further reactions (→→). BCKDK phosphorylates and inhibits the activity of the BCKD complex (T arrow). The activity of the BCKD complex is decreased in maple syrup urine disease (red ‘X’). BCAA, branched-chain amino acids; Leu, leucine; Ile, isoleucine; Val, valine; α-KG, α-ketoglutarate; Glu, glutamate; BCAT, branched-chain amino acid transaminase; BCKAs, branched-chain α-ketoacids; KIC, α-ketoisocaproate; KMV, α-keto-β-methylvalerate; KIV, α-ketoisovalerate; BCKDK, branched-chain ketoacid dehydrogenase kinase.

Branched-chain α-ketoacid dehydrogenase deficiency, commonly known as Maple Syrup Urine Disease (MSUD), is a metabolic disorder characterized by increased levels of branched-chain amino acids (BCAAs) and their respective branched-chain α-ketoacids (BCKAs) [1] (Figure 1). The three branched-chain amino acids leucine, isoleucine, and valine are unable to be synthesized by animals, and their metabolism is essential for protein synthesis and cell signaling [2]. Therefore, when BCAA metabolism is disrupted, such as in MSUD, a variety of pathologic changes arise.

The prevalence of MSUD in the United States is estimated to be 1:200,000, though some populations have much higher incidence rates. Such populations include the Mennonite population, with a reported incidence as high as nearly 1:350; the Galician population in Spain, with an incidence of 1:52,500; and the Ashkenazi Jewish population [3].

Normally, the first step in BCAA metabolism is transamination by branched-chain amino acid transaminases (BCATs) to form branched-chain α-ketoacids (BCKAs), including α-ketoisocaproate (KIC), α-keto-β-methylvalerate (KMV), and α-ketoisovalerate (KIV) for leucine, isoleucine, and valine, respectively (Figure 1). In the next rate-limiting step of the pathway, the branched-chain ketoacid dehydrogenase complex (BCKDC) catalyzes the oxidative decarboxylation of the α-ketoacids.

In the periphery, BCAA metabolism is distributed amongst many tissues. For example, transamination of BCAA to BCKA occurs at high rates in skeletal muscle. These BCKAs are then shuttled to the liver, where there are high rates of BCKDC activity. In the central nervous system (CNS), astrocytes and neurons differ in their abilities to transaminate and then decarboxylate BCAA (for review, see [4]). However, more work is required to fully understand BCAA metabolism in the brain.

MSUD arises from biallelic loss of function mutations in one of the genes that encode BCKDC subunits. Decreased BCKDC activity results in the failure of BCKAs to be oxidized into their respective end products, leading to an accumulation of BCAAs and BCKAs [5]. Due to BCAA and BCKA elevations, patients with MSUD can demonstrate acute severe ketoacidosis and neurological symptoms such as apnea, seizures, and coma as well as chronic features such as poor feeding, ataxia, motor delay, and intellectual disability due to amino acid and neurotransmitter imbalances [5,6].

Current therapies for MSUD include dietary therapy and liver transplantation. Dietary therapy requires restriction of BCAA by limiting protein in the diet and consumption of medical formulas. However, it is unclear how diet therapy affects the biochemistry of the CNS. Patients treated in this manner may still manifest a high burden of neuropsychological symptoms [5].

Liver transplantation aims to replace functional BCKDCs in the liver and to thus promote BCAA metabolism in peripheral tissue [7]. A recent study demonstrated that transplants can restore homeostasis and may arrest neurocognitive effects [5]. Unfortunately, liver transplantation was not found to improve preexisting impairments and patients are susceptible to postoperative complications and require long-term immunosuppression [7]. One long-term follow-up study of MSUD patients that had undergone treatment found that 40% of these transplantation patients have required management for acute rejection [2]. Additionally, there is evidence of similar neuropsychiatric morbidity in MSUD patients who had and had not undergone liver transplantation [8].

### 1.2. Central Nervous System Amino Acid Disruptions in MSUD

Recent studies have shown evidence of dysregulation of certain amino and organic acids in the CNS of MSUD patients, related to the disruption of BCAAs catabolism in these individuals. One study found decreased levels of glutamate and N-acetyl-aspartate (NAA) in the brain of MSUD patients [9], while another found decreased levels of phenylalanine, tryptophan, methionine, and tyrosine [5] and others found evidence of elevated lactate [10,11,12,13,14].

**Figure 2:**
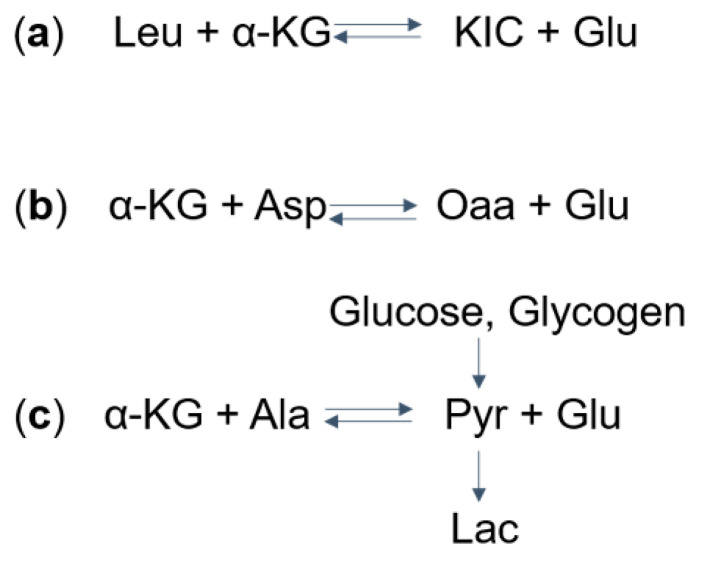
Mechanisms of (**a**) Glu, (**b**) Asp, and (**c**) Lac dysregulation in Maple syrup urine disease (MSUD): (**a**) take Leu as an example, the aminotransferase catalyzes the reversible reaction to form α-KIC and Glu from leucine and α-KG. The excess α-KIC in MSUD drives the reaction in reverse, depletes Glu, and generates α-KG. (**b**) The increased α-KG then drives the transaminase reaction to deplete Asp and to form Oaa and Glu. (**c**) Also, the α-KG can deplete Ala and produce Glu and Pyr. Pyr, which can also be formed from glucose and glycogen, is further converted into Lac. MSUD, maple syrup urine disease; Leu, leucine; α-KG, α-ketoglutarate; KIC, α-ketoisocaproate; Glu, glutamate; Asp. Aspartate; Oaa, oxaloacetate; Ala, alanine; Pyr, pyruvate; Lac, lactate.

One proposed mechanism for the decrease in glutamate and elevation in lactate levels is that, in MSUD patients, the increased levels of BCKAs (specifically α-ketoisocaproic acid) leads to reversed flux through BCAT, which normally catalyzes the conversion of BCAAs and α-ketoglutarate to BCKAs and glutamate. As a result, this reversal leads to decreased glutamate levels and increased levels of BCAAs (specifically leucine) and α-ketoglutarate [8] (Figure 2a). The excess α-ketoglutarate, along with aspartate, can then converted via aspartate aminotransferase to form oxaloacetate and glutamate [15] (Figure 2b), depleting brain aspartate levels. This excess α-ketoglutarate is also thought to drive the formation of pyruvate (a precursor to lactate) from alanine and α-ketoglutarate (Figure 2c). It was argued, though, that glutamate can be produced by the transaminase reaction from aspartate and alanine (Figure 2b,c), compensating for the reduced amount of glutamate. Another explanation for the altered levels of glutamate and lactate involves the anaplerotic role of valine and isoleucine. Valine and isoleucine significantly refill the tricarboxylic acid (TCA) cycles via succinyl-CoA. As a result, other intermediates in the TCA cycle are depleted over the time. This leads to a reduced ability of α-ketoglutarate to produce glutamate and an increased reliance on anaerobic glycolysis that produces lactate.

There is also evidence of decreased phenylalanine, tyrosine, tryptophan, and methionine in the brain of MSUD patients [5]. This is thought to arise from the excess levels of BCAAs (specifically leucine) in such patients. Leucine competes with these other amino acids for entry into the brain via the large neutral amino acid transporter (LAT1, encoded by *SLC7A5*). As leucine has the highest affinity for the transporter, excessive levels in MSUD patients reduce the ability for these other amino acids to enter the brain [8,9].

Overall, the dysregulation of these AAs may lead to brain dysfunction, predisposing MSUD patients to cognitive and psychiatric disabilities despite major clinical interventions such as liver transplant [5]. This review will therefore discuss the potential impacts of these dysregulated AAs in the brain of MSUD patients.

## 2. Potential Impacts of Decreased Brain Glutamate in MSUD

Among the amino acids that are dysregulated in the brain of MSUD patients, glutamate plays an essential role in the physiological functions of the nervous system. Glutamate is the most abundant excitatory neurotransmitter, essential for the initiation of long-term potentiation (LTP) and long-term depression (LTD). LTP and LTD are activity-dependent, persistent changes in the efficacy of neuronal synapses. It is well accepted that LTP and LTD represent cellular mechanisms underlying learning and memory [16]. Not surprisingly, extensive studies demonstrated pathogenic roles of dysfunctional glutamate pathways in learning and memory deficits (reviewed in [17]) and dementia (reviewed in [18]).

Several studies also support the role of glutamate in emotional behavior and affective disorders. A recent review has summarized multiple lines of evidence for the altered glutamate levels in the plasma, cerebrospinal fluid (CSF), and the brains of patients with depression, implicating the relevance of glutamate signaling in depressive disorders [19].

Rodent models have also implicated glutamate alterations in the pathophysiology of anxiety disorders [20,21]. Furthermore, using functional imaging, in vivo clinical data provides direct evidence of altered glutamate concentrations in emotional dysregulation. Most studies reported elevated glutamate levels across different brain regions in subjects with anxiety disorders [22,23,24,25,26], while one study found positive correlations between glutamate levels and anxiety symptoms [27]. However, others reported lower glutamate concentrations in the anterior cingulate cortex (ACC) in participants with anxiety disorders [28], and a negative correlation between glutamate concentrations in ACC and the anxiety symptoms [29]. These discrepancies may be explained by the variations in enrolled subjects and target brain regions across these studies.

The role of glutamate is also implicated in attention-deficit hyperactivity disorder (ADHD) and Obsessive-compulsive disorder (OCD). One study identified an association between a single nucleotide polymorphism (SNP) (rs6782011) in glutamate metabotropic receptor 7 (*GRM7*) gene and ADHD [30]. Corroborating this data, pathway analysis demonstrated an association between expression changes in glutamate receptor signaling genes and ADHD [31]. Another study further investigated the association of genes associated with glutamate signaling and the severity of ADHD symptoms. Although single-gene analysis failed to discover a correlation, simultaneous analysis of multiple genetic variants revealed a significant association of glutamate gene sets with the severity of hyperactive/impulsive symptoms in ADHD subjects [32]. Consistent with this finding, increased glutamate levels in ACC were positively correlated with the level of hyperactivity/impulsivity in ADHD subjects [33]. Among the studies that compared the glutamate levels in ADHD participants and healthy controls, one study reported no significant differences of glutamate concentrations within the ACC and striatum [34], yet most studies reported altered glutamate concentrations across different cerebral areas of ADHD participants as compared to healthy subjects [35,36,37,38]. OCD is another disorder that may be affected by the dysregulated glutamate (reviewed in [39]). The precise role of glutamate in OCD remains to be determined. However, several glutamate-related genes, particularly *SLC1A1* (which encodes the glutamate transporter EAAT3), have been associated with increased OCD risk (for a recent review, see [40]). Thus, the decreased glutamate levels may result in a reduced function of EAAT3 and contribute to the OCD.

Abnormal glutamatergic neurotransmission has also been implicated in the development of psychosis. Accumulating evidence confirmed alterations of glutamate metabolites in brain structures (frontal lobe, thalamus, and the associative striatum) of individuals with an elevated risk for psychosis (reviewed in [41]). A recent meta-analysis that pooled data from 28 studies identified significant lower glutamate levels in the thalamic of the high-risk psychosis group compared to the control group [42].

Moreover, glutamate, as a neurotransmitter, can affect neuronal energy metabolism. Exposure of glutamate to cortical neurons simultaneously resulted in a decreased ATP concentration and increased ADP/ATP ratios in neurons [43]. Increased oxygen consumption was also observed in glutamatergic neurons activated by electrical stimulation or glutamate exposure [43,44]. Consistent with these findings, activation of glutamate receptors resulted in a decreased local oxygen concentration [45]. This is followed by a hyperemic cerebral blood flow with a compensated elevated oxygen level in local tissue [45]. While glutamate can affect neuronal metabolism, neuronal energy consumption in the context of MSUD has not been explored.

Studies of neurological symptoms in MSUD patients remain somewhat limited. Currently, available literature showed that, despite strict diet therapy or liver transplantation that restore peripheral BCAA homeostasis, MSUD patients are at higher risk for neuropsychological impairments including cognitive deficits and mental illness (anxiety, depression, ADHD, and OCD) compared to healthy controls [9,46,47,48]. Although psychosis is commonly seen in inborn errors of metabolism [49], only a few cases of acute psychosis and hallucinations were reported in MSUD patients [50,51,52]. This might be due to the current management of MSUD, which greatly improves BCAA metabolism. So far, the majority of studies support altered glutamate in the brain of MSUD patients. However, whether altered glutamate neurotransmission is a direct cause of the neurological symptoms in MSUD requires further investigation.

## 3. Potential Impacts of Decreased Brain N-Acetylaspartate and Aspartate in MSUD

N-Acetylaspartate (NAA) is the second-most abundant amino acid in the human brain [53]. Several studies have demonstrated NAA depletion in the brain of MSUD patients during acute metabolic decompensation [10,13,14,54]. When compared to the healthy controls, MSUD subjects exhibited significantly lower concentrations of NAA in the brain [9,12]. Notably, lower brain NAA levels in MSUD patients were correlated with higher severity of anxiety, depression, and ADHD symptoms [9].

The metabolism of NAA and its derivative N-acetylaspartylglutamic acid (NAAG) involves neurons, astrocytes, and oligodendrocytes, a tricellular metabolic sequence [53]. The proposed roles for NAA include neuronal osmoregulation, axonal-glial signaling, and lipid synthesis in oligodendrocytes (for review, see [55]). Importantly, NAA catabolism in the oligodendrocytes provides the acetyl group essential for fatty acid synthesis in myelin lipids. As a consequence, NAA deficiency in MSUD may result in defective myelination. Indeed, myelination differences are frequently observed in MSUD patients on magnetic resonance imaging (MRI) scans [56,57,58,59,60,61]. Additional evidence from histological and electrophysiological data also demonstrated myelin destruction and axonal degeneration in a MSUD case [62]. Since the destruction of myelin integrity may be involved in the pathogenesis of depression (reviewed in [63]) and ADHD (reviewed in [64]), the lower NAA concentrations in MSUD may influence myelin sheath and correlate to the higher severity of depression and ADHD symptoms in MSUD patients.

Accumulating evidence also supports a pathogenic role of NAA deficiency in epilepsy. Severely reduced NAA was reported in patients with homozygous aspartate-glutamate carrier 1 (AGC1) mutations, which cause infantile epilepsy [65,66,67,68]. Based on the fact that AGC1 mediates the efflux of aspartate from neuronal mitochondria to cytoplasm, it has been proposed that the deficient function of AGC1 leads to impaired transportation of aspartate to the cytoplasm, where can be metabolized to NAA [69]. As noted in the previous section, there are decreased levels of NAA in the brain of MSUD patients [9]. This may result from reduced neuronal density and/or compromised synthesis from aspartate, the only precursor of NAA.

So far, the levels of aspartate in the brain of MSUD patients have not been determined. However, experimental data demonstrated aspartate depletion with exposure to KIC in vitro [70]. In vivo studies also demonstrated significantly lower aspartate levels in the brain tissue of an intermediate MSUD mouse (iMSUD) [71] and neonatal MSUD claves [72], compared to control animals.

Aspartate is a nonessential amino acid that can be synthesized via the transamination of oxaloacetate. There are two forms, L-aspartate (L-Asp) and D-aspartate (D-Asp), which can be converted from L-Asp via the enzyme D-aspartate racemase. Both of the L- and D-forms are present in the brain and modulate neuronal activity. L-Asp has been proposed as a major excitatory neurotransmitter; for a review of the vesicular transportation and co-transmission of L-Asp, please see [73]. Recent evidence also demonstrates a role of D-Asp in the CNS. Studies indicate that D-Asp binds and activates N-methyl-D-aspartate receptors (NMDARs), modulates LTP and synaptic plasticity, and is involved in the synthesis and release of multiple hormones (reviewed in [74]).

In addition to its role as a neurotransmitter, aspartate is an essential metabolite in the malate-aspartate shuttle (MAS). In the CNS, MAS is mainly expressed in neurons, with low activity in astrocytes [75]. MAS plays a prominent role in neuronal mitochondrial respiration. In primary neurons, deficiency in MAS results in a 46% drop in mitochondrial respiration [76]. Hence, the decreased aspartate in MSUD may affect oxidative phosphorylation and adenosine triphosphate production in neurons (reviewed elsewhere [15]). Furthermore, studies reported that MAS deficits are associated with infantile-onset epileptic encephalopathies [65,66,67,68,77,78] and autism [79,80,81,82]. In short, reduced aspartate levels in the CNS of MSUD patients may decrease MAS flux and contribute to neurologic symptoms in MSUD patients.

## 4. Potential Impacts of Increased Brain Lactate in MSUD

Several studies detected an abnormal lactate peak in the brain of MSUD patients [10,11,12,13,14]. In addition to its role as a vital metabolite, research in recent decades suggests that lactate is an important signaling molecule in the brain. In the locus coeruleus, a nucleus primarily consisting of noradrenergic neurons, the neuronal responses are dependent on the local concentrations of lactate [83]. Lactate from astrocytes or exogenous administration stimulates the neuronal release of norepinephrine, which is a principle neurotransmitter involved in stress, panic, anxiety, and depression disorders [83]. Clinical studies further support a role for brain lactate in panic disorder. Functional imaging studies revealed lactate elevation following neural activation in the brain of healthy subjects [84,85,86]. Moreover, the elevated brain lactate was significantly greater in subjects with panic disorder as compared to controls [86]. While elevated brain lactate can occur in MSUD patients [87], it remains to be determined whether the increased brain lactate derives directly from the brain or peripheral tissue through transportation across the blood–brain barrier (BBB). Notably, panic disorder, which is very prevalent in the MSUD population [5], may not be reversed by liver transplantation [88]. Therefore, brain-derived lactate may play a primary, if not exclusive, role in the pathogenic mechanism of panic disorder in MSUD.

Three main pathways account for the brain-derived lactate in MSUD (Figure 2c). It is possible that, in MSUD, pyruvate fluxes into accumulated lactate rather than entering the citric acid cycle. As described in the previous section, α-ketoacids from metabolized BCAAs drive the production of pyruvate through transaminase reactions. Pyruvate can also be formed through glycolysis and glycogenolysis. In all three processes, pyruvate can be further converted into lactate for continued glycolysis or feed the citric acid cycle for aerobic respiration in the mitochondria. The mitochondrial respiration is inhibited in MSUD. Supporting this notion, in vitro studies in the rat cortex revealed that BCAAs or their α-ketoacids derivatives significantly lowered the activities of the mitochondrial electron transport chain complexes [89,90]. Experimental evidence in the rat brain mitochondria additionally showed that the α-ketoacid from metabolized leucine inhibits the mitochondrial import of pyruvate for aerobic oxidation [91,92]. Importantly, as discussed above, the MAS, which accounts for 46% of the mitochondrial respiration in neurons [76], may also be compromised due to aspartate deficiency in MSUD.

It is noteworthy that, although it only accounts for 2% of the body’s mass, the brain accounts for 20% of total oxygen metabolism [93]. To compensate for the reduced energy production from oxidative respiration and to meet the high-energy demand of the brain in MSUD, cells may instead promote utilization of glucose via glycolysis. Consistent with this idea, MSUD patients and animal models demonstrate lower plasma glucose levels as compared to controls [94,95,96,97,98]. Early studies suggested a role for leucine in MSUD-associated hypoglycemia. It was proposed that leucine stimulated release of insulin from pancreatic beta cells, thus promoting glucose utilization [99]. However, recent experimental evidence suggests an insulin-independent regulation of glucose in the brain of MSUD. In studies of the in vitro culture of rat cortical slices, incubation with BCAAs or their α-ketoacids derivatives significantly increased glucose uptake by the cultured cortical slices [89,90]. Therefore, further studies characterizing aerobic respiration and glycolysis in MSUD may reveal the etiology of elevated lactate in MSUD. In particular, studies that address the brain-specific role in energy metabolism in MSUD are necessary.

## 5. Potential Impacts of Decreased Brain Phenylalanine, Tyrosine, Tryptophan, and Methionine in MSUD

**Figure 3:**
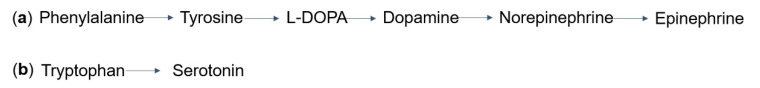
Aromatic amino acids are the biosynthesis precursors to (**a**) dopamine, norepinephrine, epinephrine, and (**b**) serotonin.

The concentrations of phenylalanine, tyrosine, tryptophan, and methionine in the brain are estimated to be lower in MSUD patients as compared to healthy controls [5]. These AAs are precursors for several neurotransmitters (Figure 3). For example, aromatic AAs (phenylalanine, tyrosine, and tryptophan) are the biosynthetic precursors to the monoamine neurotransmitters including dopamine, norepinephrine, epinephrine, and serotonin. It has been well established that deficiency of these monoamine neurotransmitters are related to neuropsychiatric disorders including major depressive disorder [100], anxiety disorder [101], ADHD [102], and movement disorders [103]. Thus, deprivation of the aromatic AAs may play a central role in the neurological symptoms of MSUD. Evidence supporting the role of monoamine neurotransmitters in MSUD includes reports of decreased monoamine neurotransmitter concentrations in an intermediate MSUD mice model (iMSUD). Specifically, the levels of serotonin, dopamine, and dopamine intermediate 3,4-dihydroxyphenylacetate were significantly reduced in the iMUSD brain as compared to the wildtype controls [104,105]. The role of monoamine neurotransmitters in the human MSUD brain remains poorly understood. Interestingly, the dysregulations of the aromatic AAs and monoamine neurotransmitters are involved in the pathophysiology of Phenylketonuria (PKU), which is characterized by increased phenylalanine and decreased tyrosine, serotonin, dopamine, and norepinephrine [106]. Untreated PKU patients can exhibit similar neurological deficits as MSUD, including seizures and intellectual disability [107]. These neurological damage in PKU can be prevented if phenylalanine-free diet is initiated at an early age [107], thus suggesting a pathological role of dysregulated aromatic AAs in the neurological damage.

Methionine is crucial for the synthesis of cysteine and glutathione, both of which are essential antioxidants in protecting against the formation of reactive oxygen species (ROS) (reviewed in [108,109]). Decreased brain methionine in MSUD patients may result in depletion of antioxidants, with a consequent elevation of cellular vulnerability towards oxidative stress. Indeed, animal studies have suggested a role of oxidative damage in the neuropathology of MSUD. One group demonstrated that chronic exposure to high concentrations of BCAAs resulted in impaired learning and memory in rats [110,111]. Interestingly, these impairments can be fully reversed by antioxidant treatment, suggesting a role of oxidative stress in BCAA-induced cognitive dysfunctions [110,111]. Studies from another group provided further evidence supporting BCAA-induced oxidative stress. In vitro studies demonstrated that incubation of the branched-chain α-ketoacids with rat cortex stimulated lipid peroxidation and inhibited antioxidant defenses [112]. Consistent with these findings, an imbalance between oxidative damage and antioxidant defense was observed in a chemical-induced MSUD in vivo model. Rats were repeatedly administrated with BCAAs subcutaneously, and oxidative parameters in the cortex were evaluated. Indicators of oxidative damage such as lipid and protein peroxidation were significantly higher in the cortex of MSUD rats compared to the control rats [113,114]. Conversely, brain antioxidant systems were significantly inhibited, including decreased activities of antioxidant enzymes and lower concentrations of glutathione [113,114].

We identified only one study investigating oxidative damage in MSUD patients. The authors evaluated plasma levels of oxidative stress biomarkers, such as protein carbonyl and malondialdehyde [115,116]. These biomarkers were significantly higher in MSUD patients as compared to healthy controls, suggesting elevated oxidative stress in the peripheral tissue of MSUD patients [1]. However, the role of oxidative stress in the brain of MSUD patients has not been studied.

As mentioned, few studies have explored the levels of the monoamine neurotransmitters and potential oxidative damage in the brain of MSUD patients. This may partially be due to technical limitations. For example, the dopamine signals acquired by in vivo magnetic resonance spectroscopy (MRS) are too low for quantitative analysis [117]. Therefore, several different approaches have been tried. For example, positron emission tomography (PET) has been used to image tracers that target presynaptic and postsynaptic dopaminergic neurons. While this measurement facilitates characterization of the dopaminergic system, it may not provide direct evidence of altered dopamine concentrations in the brain. An alternative approach would determine the dopamine concentrations in the CSF [118], which requires an invasive procedure.

In summary, evidence suggests that abnormalities in aromatic AAs, the related monoamine neurotransmitters, methionine, and antioxidants cysteine and glutathione may contribute to BCAA-induced neurological symptoms in MSUD. However, further studies that define the alterations of these metabolites in the brain of MSUD are required. Animal models may provide invaluable tools to study these metabolites in the brain of MSUD. Meanwhile, in vivo detection in MSUD patients may be achieved via novel imaging or diagnostic approaches.

## 6. Altered BCAAs Metabolism in Other Neurological Disorders

As mentioned above, disturbances in BCAAs may contribute to neurologic dysfunction in MSUD, including cognitive impairment, depression, anxiety, AHDH, panic disorder, and epilepsy. In this section, we will briefly discuss evidence supporting the role of BCAA alterations in other neurological disorders.

### 6.1. BCAA and Autism Spectrum Disorder (ASD)

Autism is a neurodevelopmental disorder that significantly impairs communication and behavior. A number of recent publications have demonstrated a link between BCAA dysregulation and increased risk for ASD. Likely pathogenic variants in the genes including the LAT1 and LAT2 transporters, which import BCAA into the brain, were enriched in cohorts of individuals with ASD [119,120]. Furthermore, pathogenic variants in branched-chain ketoacid dehydrogenase kinase (BCKDK) lead to autism [121,122]. BCKDK phosphorylates and inhibits the activity of the BCKDC, which catalyzes the rate-limiting reaction in the BCAA catabolism (Figure 1). Hence, inactivating mutations in BCKDK leads to constitutively activated BCKDC, leading to substantial reductions in BCAAs and BCKAs. Furthermore, in patients without an identified molecular etiology of their autism, lower BCAA levels were observed in the plasma [122,123,124], urine [122,125], and CSF [122,126]. These lines of evidence indicate that lower levels of BCAA may contribute to ASD. This theory is further supported by experimental data, demonstrating that intracerebroventricular delivery of BCAAs ameliorates autism-related phenotypes in mouse models [120]. Interestingly, one study also identified loss of function BCAT mutations in patients with autism [127]. Since BCAT activity generates BCKAs from BCAAs (Figure 1), as expected, these patients had higher BCAA levels in plasma [127]. Hence, these studies support the relevance of dysregulated BCAA and BCKA in autism.

### 6.2. BCAA and Alzheimer’s Disease (AD)

AD is a neurodegenerative disease and the most common cause of dementia in the elderly. It is characterized by the accumulation of extracellular amyloid β and intracellular hyperphosphorylated tau. Recent advances suggest a potential link between AD pathogenesis and BCAAs.

Altered levels of circulating BCAAs were identified in several animal models of AD. When compared to controls, APPswe/PS1dE9 and 3xTg AD (APPswe/PS1m146v/TauP301L transgenic) mouse displayed higher plasma levels of BCAAs at certain ages [128,129,130]. Studies of BCAA in human AD patients are limited. One study reported decreased serum valine levels in the AD patients as compared to controls [131]. However, another study pooled AD subjects with or without diabetes and identified increased plasma BCAA levels in the AD group compared to the control [129]. The role of the altered BCAA levels in AD pathology remains to be determined. In prospective studies, lower serum concentrations of valine were associated with a higher risk for AD, worse cognitive function, and faster cognitive declines [132]. Furthermore, a meta-analysis that pooled eight cohort studies identified an inverse associations between circulating BCAAs and AD [133].

Experimental evidence from animal studies suggests that dietary BCAA consumption may promote the development of AD. A high-BCAA diet worsened Tau pathology and cognitive performance in 3xTg mice yet did not significantly alter outcomes in wild-type mice. Conversely, reduction in BCAA consumption improved performance on memory tasks in the 3xTg mouse model [134]. Collectively, studies of BCAAs in AD are far from complete and it is hard to draw a conclusion based on current evidence. Nevertheless, the early data supports a possible role for BCAAs in AD pathogenesis or progression.

### 6.3. BCAA and Huntington’s Disease (HD)

Movement disorders have been observed in MSUD patients, particularly those with metabolic decompensations in childhood [47,135,136]. Interestingly, BCAA disturbances have also been linked to neurodegenerative movement disorders including Huntington’s disease (HD) and Parkinson’s disease (PD).

The pathophysiology of HD is not fully understood, but the CAG-repeat length is inversely correlated with the age of onset [137]. In the context of HD, several studies reported decreased BCAA levels in the plasma of HD patients as compared to healthy controls [138,139,140,141]. The decreased BCAA levels were also observed in the CSF of HD subjects [138]. In line with this evidence, another study identified reduced valine in both the striatum and frontal lobe of HD patients as compared to controls [142]. Remarkably, plasma BCAA levels were negatively correlated with the severity of motor dysfunction [139] and the number of CAG repeats in HD [139,140]. The decreased serum BCAA levels may be a consequence of hypermetabolic states and high-energy demands in HD, as the lower BCAA levels were correlated with weight loss in HD patients [139].

BCAA abnormalities are also observed in HD animal models. In cortical and striatal slices from the HD mouse brain, there was an enhanced metabolic flux of isoleucine into the tricarboxylic acid (TCA) cycle as compared to controls. While several studies support the presence of BCAA alterations in HD patients, more work is needed to elucidate the mechanisms driving these changes.

### 6.4. BCAA and Parkinson’s Disease (PD)

Several studies have demonstrated BCAA abnormalities in PD; however, studies have yielded conflicting results. In studies of CSF from PD patients, some groups have found reduced BCAA concentrations [143,144] while others have identified increased BCAA concentrations as compared to controls [145]. Studies also examined the peripheral BCAA levels in PD patients. When compared to healthy controls, PD participants displayed higher BCAA levels in the plasma [143,144,146], saliva [147], and urine [148]. In one study that explored the potential correlations of serum BCAAs and PD development, none of the serum BCAAs were associated with the likelihood of PD [149]. Thus, although some reports identified altered BCAAs in PD, additional studies are required to fully define this relationship.

## 7. Conclusions

In conclusion, current studies suggest that alterations in BCAA metabolism can induce neurologic dysfunction. Maintaining normal BCAA metabolism in the brain itself is crucial to neurologic health, as evidenced by the fact that current MSUD therapies which significantly improve peripheral biochemistry fail to prevent neuropsychiatric symptoms. Dietary therapy and liver transplantation significantly improve survival rates and decrease the frequency of metabolic crises but cannot prevent all CNS manifestations of disease [9,12]. Future studies are needed to establish how disruptions of BCAA metabolism in the brain lead to neurological dysfunction in order to both advance therapy development for MSUD and open novel avenues of drug development for a variety of neurological disorders.
